# Nasopharyngeal viral load predicts hypoxemia and disease outcome in admitted COVID-19 patients

**DOI:** 10.1186/s13054-020-03244-3

**Published:** 2020-09-01

**Authors:** Amir Shlomai, Haim Ben-Zvi, Ahinoam Glusman Bendersky, Noa Shafran, Elad Goldberg, Ella H. Sklan

**Affiliations:** 1 Department of Medicine D, Rabin Medical Center, Beilinson Hospital, Petah Tikva, Israel; 2 The Sackler Faculty of Medicine, Tel Aviv University, Tel Aviv, Israel; 3 Microbiology Laboratory, Rabin Medical Center, Beilinson Hospital, Petah Tikva, Israel; 4 Department of Medicine F, Rabin Medical Center, Beilinson Hospital, Petah Tikva, Israel; 5 Department of Clinical Microbiology and Immunology, The Sackler Faculty of Medicine, Tel Aviv University, Tel Aviv, Israel

**Keywords:** Viral load, SARS-CoV-2, COVID-19, Hypoxemia

## Introduction

The SARS-CoV-2 pandemic imposes an unprecedented burden on hospitals treating coronavirus disease 2019 (COVID-19) patients. Thus, clinical parameters accurately predicting disease outcome are needed. Here, we identified a correlation between viral load measured around admission, lung inflammation, and disease outcome. Similarities and differences between related studies are discussed.

## Methods

Viral loads of COVID-19 patients admitted to Rabin Medical Center between March 16 and July 23, 2020, were analyzed (*n* = 170, females 42%, age 62 (IQR 46–73), hospitalization length 7.5 days (IQR 3–13)). Clinical and demographic data were collected from the patients’ electronic medical records. Nasopharyngeal samples were collected and transferred to the microbiology laboratory for testing. Quantitative RT-PCR was performed using the Allplex™ 2019-nCoV Assay (Seegene). Presented are Ct values of the nucleocapsid N gene from the first test performed for each patient. Simple linear regression of clinical parameters against the viral load was fitted to the data to assess the association between viral and clinical parameters using GraphPad Prism. Adjusted odds ratio (OR) of mechanical ventilation and mortality were calculated for each significant variable with 95% confidence intervals (CI) using SPSS, version 26.

## Results

**Fig. 1:**
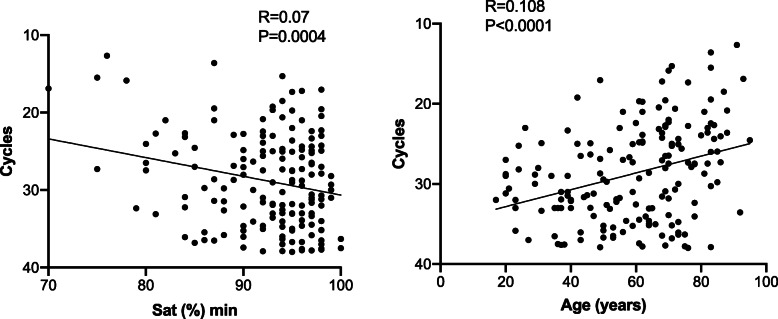
Viral load is associated with minimal saturation values and age. The relationship between Ct values from the nucleocapsid gene, minimal saturation values and age (*n* = 170). Similar results were obtained with the Ct for the envelope gene. (R=R squared, pearson's correlation test)

**Fig. 2:**
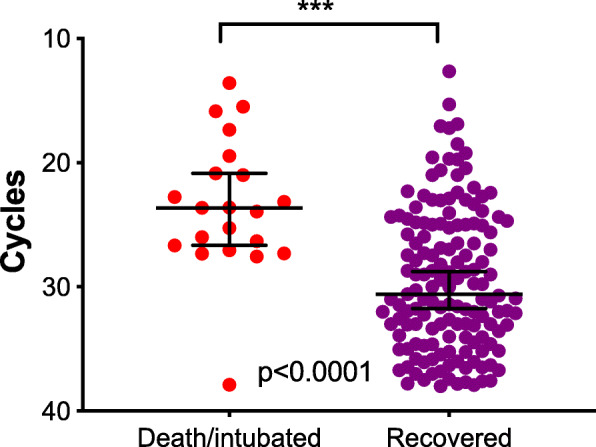
Viral load is associated with disease outcome. A scatter plot presenting the Ct values (median, 95% CI) of admitted COVID-19 patients according to clinical outcome

Cycle threshold values of the first test performed for all admitted COVID-19 patients were correlated with the patients’ clinical parameters. Among the parameters tested (lowest values of albumin, lymphocyte count, blood oxygen saturation (BOS) and systolic blood pressure, peak levels of lactate dehydrogenase (LDH), C-reactive protein (CRP), ferritin, white blood cell count, and fever), only BOS_min_ (*R* = 0.07, *p* = 0.0004) showed significant correlation (Fig. 1). Interestingly, patients’ age was also significantly correlated with viral load (Fig. 1). Non-survivors and mechanically ventilated patients (*n* = 21) had a significantly higher viral load (8-folds, Ct = 23.43 ± 5.38) compared to surviving non-intubated patients (*n* = 149, Ct = 29 ± 5.55, *t* test *p* < 0.0001, Fig. 2). A multivariate analysis adjusted for age, gender, and BOS_min_ revealed that low viral load was independently associated with reduced risk for mechanical ventilation and mortality (OR = 0.90, 95% CI 0.81–0.99, *p* = 0.046). Furthermore, BOS and patients’ age were also independently associated with mechanical ventilation and death (OR = 0.91, 95% CI 0.84–0.98, *p* = 0.009 for BOS and OR = 1.05, 95% CI 1.004–1.097 for age).

## Discussion

Our results show a direct link between nasopharyngeal viral load and hypoxemia, as well as worse disease outcomes in admitted patients with COVID-19. Previous studies tested the association between viral load and survival. In agreement with our results, a study of 678 admitted patients in New York found that 35.0% of patients with a high viral load on admission died, compared to 6.2% of patients with low viral loads [1]. In an older age cohort (*n* = 48, age 67–97) from Belgium, clinical frailty scale, LDH, and viral load predicted survival [2]. Intensive care unit (ICU) admission also positively correlated with detectable viral RNA in anal swabs [3]. In contrast, a Swiss study found no correlation between viral load and disease outcome. The study compared the viral load of patients admitted to the ICU (*n* = 48) to patients treated in a screening unit (*n* = 723) [4]. It is not clear, however, whether these patients were later admitted, intubated, or survived. A different New York study (*n* = 205) found no association between viral load and disease severity parameters. However, this study mainly compared non-hospitalized to hospitalized patients [5]. Several markers were associated with COVID-19 severity, the most accepted is IL-6 [6]. However, IL-6 is not routinely tested at admission and might reflect other inflammatory conditions. Thus, in spite of differences in test kits and procedures between different laboratories and institutions, viral load might provide a rapid screening tool for COVD-19 severity among admitted patients.

## Data Availability

The dataset supporting the conclusions of this article is available from the authors upon request.

## Abbreviations

SARS-CoV-2: Severe acute respiratory syndrome coronavirus 2
COVID-19: Coronavirus disease 2019
IQR: Interquartile range
Ct: Cycle threshold
RT-PCR: Reverse transcription polymerase chain reaction
OR: Odds ratio
CI: Confidence interval
LDH: Lactate dehydrogenase
BOS: Blood oxygen saturation
CRP: C-reactive protein
ICU: Intensive care unit
